# The Effect of Various Types of Mechanical and Chemical Preconditioning on the Shear Bond Strength of Orthodontic Brackets on Zirconia Restorations

**DOI:** 10.1155/2017/6243179

**Published:** 2017-01-11

**Authors:** Jihun Kim, Chanhee Park, Jeong-Sub Lee, Jungho Ahn, Yoon Lee

**Affiliations:** ^1^Department of Pediatric Dentistry, Wonju Severance Christian Hospital, Wonju College of Medicine, Yonsei University, Wonju, Republic of Korea; ^2^Department of Orthodontics, Wonju Severance Christian Hospital, Wonju College of Medicine, Yonsei University, Wonju, Republic of Korea; ^3^Department of Conservative Dentistry, Wonju Severance Christian Hospital, Wonju College of Medicine, Yonsei University, Wonju, Republic of Korea

## Abstract

The purpose of this study was to investigate the combined effect of mechanical and chemical treatments on the shear bond strength (SBS) of metal orthodontic brackets on zirconia restoration. The zirconia specimens were randomly divided into 12 groups (*n* = 10) according to three factors: AL (Al_2_O_3_) and CO (CoJet™) by sandblasting material; SIL (silane), ZPP (Zirconia Prime Plus), and SBU (Single Bond Universal) by primer; and N (not thermocycled) and T (thermocycled). The specimens were evaluated for shear bond strength, and the fractured surfaces were observed using a stereomicroscope. Scanning electron microscopy images were also obtained. CO-SBU combination had the highest bond strength after thermocycling (26.2 MPa). CO-SIL showed significantly higher SBS than AL-SIL (*p* < 0.05). CO-ZPP resulted in lower bond strength than AL-ZPP before thermocycling, but the SBS increased after thermocycling (*p* > 0.05). Modified Adhesive Remnant Index (ARI) scoring and SEM figures were consistent with the results of the surface treatments. In conclusion, CO-SBU, which combines the effect of increased surface area and chemical bonding with both 10-MDP and silane, showed the highest SBS. Sandblasting with either material improved the mechanical bonding by increasing the surface area, and all primers showed clinically acceptable increase of shear bond strength for orthodontic treatment.

## 1. Introduction

With the increasing popularity of social networking sites and the use of high resolution digital images, maintaining the esthetics of the maxillofacial area is becoming increasingly important in the field of dentistry [1, 2]. Adolescents are especially prone to dental caries and trauma and are likely to undergo orthodontic treatment, and esthetic restorations are becoming more popular among this age group [2, 3]. Among esthetic restorative materials, zirconia is being widely used on both deciduous and permanent dentition [3, 4]. As a result, additional studies evaluating esthetic materials in various clinical situations are required [5].

Previous studies evaluating zirconia are mostly limited to bond strengths between zirconia restorations and tooth structure [5], and these bonds are vastly different from those between metal brackets and zirconia restorations. Since the bond formed in this case is with an existing zirconia restoration, preconditioning is limited to chair-side methods. Available preconditioning methods include air-abrasion and primer application, and the expected time duration and debonding force are based on the orthodontic treatment rather than on permanent bonding of restorations [6, 7].

Zirconia crystals change phase depending on the temperature. Zirconia has a stable monoclinic crystalline structure up to 1107°C. Between 1170°C and 2370°C, it is in a tetragonal phase. Above this temperature, it takes on a cubic form until it reaches a temperature of 2680°C [8]. Depending on outside stress or the treatment methods used, the tetragonal structure may change to a monoclinic crystalline structure. Transformation toughening occurs with such a transition, and with consequent volume expansion of zirconia, crack propagation is inhibited. Zirconia therefore shows superior fracture and flexural strength as compared to conventional ceramic restoratives [9].

Compared to tooth enamel, zirconia exhibits a lower bonding strength with orthodontic brackets and, consequently, surface preconditioning such as sandblasting or primer application is necessary [5, 10]. However, even in cases of stabilized zirconia, stress and heat treatment during sandblasting or manufacturing may cause phase transition and residual stress in the material. In conventional sandblasting, Al_2_O_3_ particles are used to abrade the zirconia surface resulting in the formation of a microretentive surface [11]. Previous studies show that when silane or zirconia primer is applied to zirconia, a chemical bond is achieved, improving the bond strength [12, 13].

Both zirconia primer and some universal bonds contain 10-methacryloyloxydecyl dihydrogen phosphate (10-MDP). Because 10-MDP is hydrophobic, it exhibits a relatively stable bond and is durable [5, 14, 15]. It also forms a chemical bond with the zirconia surface, resulting in a strong resin bond. When zirconia is abraded with silica-coated alumina particles such as CoJet™, an even stronger bond may be achieved [16, 17].

Both temperature changes and moisture content of the oral environment affect bond strength, and thermocycling studies were performed to evaluate their effects on the bond strength between zirconia and the metal bracket [14, 18]. Other aging studies include long-term water storage to investigate its effect on bond strength [19, 20].

Shear bond strength testing has often been used to analyze the state of the bond between an orthodontic bracket and a tooth restoration. Fracture pattern analysis is often performed at the same time [21, 22].

Therefore, the purpose of this study was to investigate the combined effect of two mechanical treatments (sandblasting by Al_2_O_3_ and CoJet) and three chemical treatments (silane, zirconia primer, and Single Bond Universal) on the shear bond strength (SBS) of metal orthodontic brackets on zirconia restoration and to evaluate the effect of thermocycling. The fracture pattern was analyzed by modified ARI (Adhesive Remnant Index as described by Artun and Bergland) through a stereomicroscope [22, 23], and scanning electron microscopic images were analyzed to investigate the surface change of zirconia.

## 2. Materials and Methods

**Figure 1:**
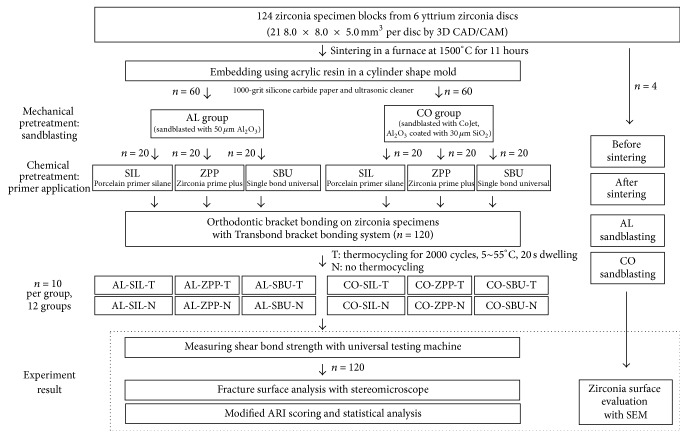
Schematic diagram of experiment procedures.

The materials and equipment used in this study are shown in Tables 1, 2, and 3 and the schematic description of the procedures is shown in Figure 1.

### 2.1. Specimen Preparation

**Figure 2:**
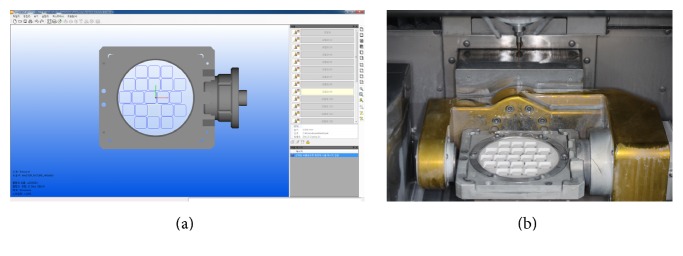
Zirconia preparation by 3D CAD/CAM. (a) Computer-Aided Design with software (hyperDENT, Follow-Me Dental Engineering, Germany). (b) Computer-Aided Milling (CAM) machine (Trione M5, DIO, Korea).

A total of 124 zirconia blocks were prepared from six yttrium oxide-stabilized zirconia discs (Z-Brick: ZirPremium-WDS10-NP(+) A2, LOT#1160222EA2Y2, ACUCERA, Korea) of 10T thickness, which are used for dental restorations. The specimens were fabricated using Computer-Aided Design (CAD) software (hyperDENT, Follow-Me Dental Engineering, Germany) to create 21 8.0 × 8.0 × 5.0 mm^3^ rectangular specimens per disc. A Computer-Aided Milling (CAM) machine (Trione M5, DIO, Korea) was used to cut the discs (Figure 2). All zirconia blocks were sintered in a furnace (DuoTron Pro, B&D Dental Technologies, USA) at 1500°C for 11 hours. This was performed by one lab technician according to the manufacturer's recommendations. The zirconia blocks were embedded in a cylinder shaped mold using epoxy resin (powder: Vertex, LOT#XU272PO4, Vertex Dental, Netherlands; liquid: KM-88 self-curing resin liquid, Kmaster Products, Korea) with the surface for bracket adhesion facing up. The cylindrical mold had a diameter of 30 mm and a height of 15 mm to match the specifications of the universal testing machine.

### 2.2. Group Allocation and Surface Treatment

To create uniform surfaces on the zirconia blocks, the surfaces were ground with 1000-grit silicone carbide paper. The blocks were then placed in an ultrasonic cleaner. The specimens were randomly allocated to 12 groups of 10 each depending on the surface treatment method used and whether thermocycling was performed (Table 1). Four additional specimens were designated for scanning electron microscopy analysis.

#### 2.2.1. Mechanical Surface Treatment through Sandblasting

To evaluate mechanical surface pretreatment through sandblasting, the specimens were divided into two groups: the AL group, which was sandblasted with 50 *μ*m Al_2_O_3_ (Blasting Medium for crowns and bridges, LOT#444262, Dentaurum, Germany) and the CO group, which was treated with Al_2_O_3_ coated with 30 *μ*m SiO_2_ (CoJet Sand, 3M ESPE, St. Paul, MN, USA) (Table 2). Sandblasting was performed on all zirconia specimens by a single investigator for 10 sec at 40 psi pressure from a 10 mm distance. After sandblasting, the particles remaining on the zirconia block surfaces were rinsed away with water. The blocks were then placed in an ultrasonic cleaner (Mujigae, Seongdong, Korea) and dried with a tooth surface dryer (CLEAN WARMER III tooth surface dryer, Samjin, Korea) for 20 sec.

#### 2.2.2. Chemical Zirconia Surface Pretreatment

After sandblasting, all specimens were treated with one of three primers: SIL (silane), ZPP (Zirconia Prime Plus), or SBU (Single Bond Universal) (Tables 2 and 3). The primers were applied according to the manufacturers' recommendations and they were either dried with tooth surface dryer or light-cured at 1400 mW/cm^2^ for 15 sec (Valo Cordless LED Curing Light, Ultradent Products).

### 2.3. Bonding of Orthodontic Brackets

Mandibular anterior orthodontic metal brackets were bonded (LOT#C475 and C575, Tomy Inc., Tokyo, Japan) on 120 of the zirconia specimens after mechanical and chemical pretreatment. Orthodontic adhesive primer (Transbond™ XT primer, LOT#N731433, 3M Unitek, Monrovia, CA, USA) was applied to the specimen, the excess was removed by a dryer, and it was light-cured (Valo Cordless LED Curing Light, Ultradent Products). Afterwards, orthodontic resin paste (Transbond XT primer, LOT#N636253, 3M Unitek, Monrovia, CA, USA) was positioned using digital pressure and the excess was removed with an explorer. The bracket was light-cured from all four directions at a 45-degree angle and a 10 mm distance. Light curing was performed at 1400 mW/cm^2^ for 15 sec each time. The base of bracket measured 9.81 mm^2^ according to the manufacturer. The bracket-bonded specimens were stored at 100% relative humidity for 7 days and then were further subdivided based on whether thermocycling was to be done or not.

### 2.4. Thermocycling

The specimens were divided into two groups based on whether thermocycling was to be done or not: no thermocycling (N) group and thermocycling (T) group. Thermocycling (Thermal Cycling System, Hangil Technics, Korea) was performed for 2000 cycles between 5°C and 55°C with 20 sec dwell time.

### 2.5. Shear Bond Strength Measurement

**Figure 3:**
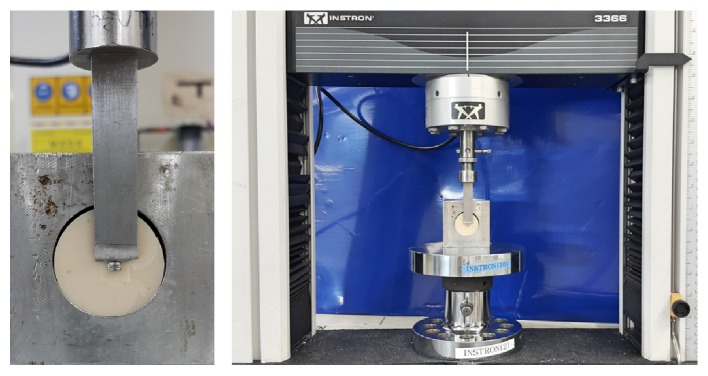
Equipment setup for measuring shear bond strength (Model 3366; Instron® Co.).

All specimens were stored at 100% relative humidity for 24 h. The bracket-bonded resin molds were positioned on the Universal testing machine (Model 3366; Instron® Co., Norwood, MA, USA), and the shear bond strength was measured at a crosshead speed of 1 mm/min (Figure 3).

### 2.6. Analysis of Fractured Surface

**Figure 4:**
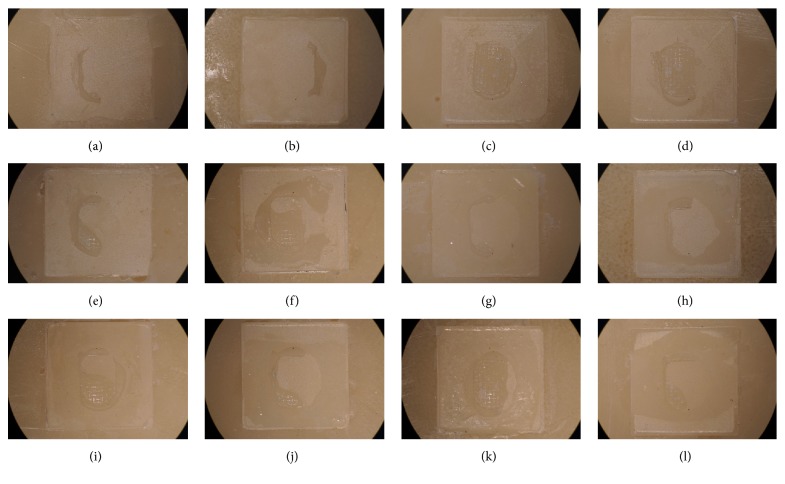
Stereomicroscopic images (12x magnification) of debonded zirconia surface. The representative images from the 12 groups were selected by modified ARI score. ((a), (b), (c), and (d)) Silane application on zirconia ((a) AL-SIL-N, (b) AL-SIL-T, (c): CO-SIL-N, and (d) CO-SIL-T); ((e), (f), (g), and (h)) Zirconia Prime Plus application on zirconia ((e) AL-ZPP-N, (f) AL-ZPP-T, (g) CO-ZPP-N, and (h) CO-ZPP-T); ((i), (j), (k), and (l)) Single Bond Universal application on zirconia ((i) AL-SBU-N, (j) AL-SBU-T, (k) CO-SBU-N, and (l) CO-SBU-T).

Following shear bond strength measurement, all specimens were observed under a stereomicroscope to analyze the fracture pattern. In addition, the remaining resin on the specimen surface was scored according to the modified Adhesive Remnant Index (ARI) by three different investigators. When there was more than 2-point difference between them, the score was reevaluated until an agreement was reached between the investigators (Table 4). One representative specimen from each group was recorded using a stereomicroscope (SZ-PT 40, OLYMPUS, Japan) at a magnification of 12x (Figure 4).

### 2.7. Scanning Electron Microscopy of Zirconia Surface

**Figure 5:**
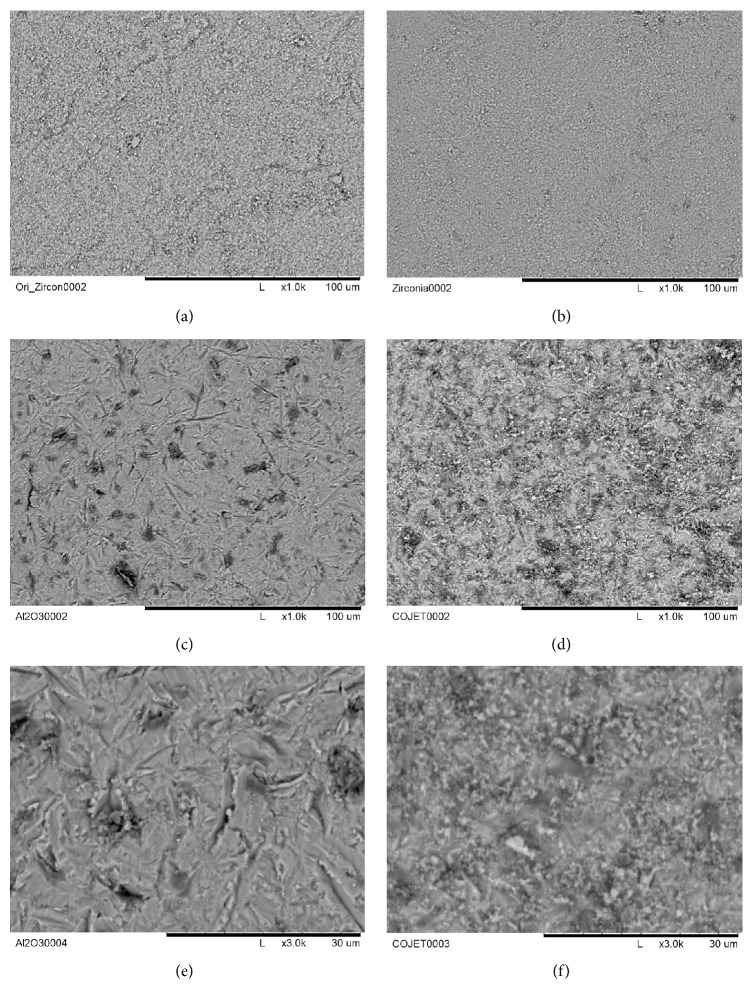
Scanning electron microscopy images of zirconia surface (magnification 1000x). (a) Zirconia surface before sintering; (b) zirconia surface after sintering; (c) Al_2_O_3_ sandblasting after sintering; and (d) CoJet (SiO_2_-coated Al_2_O_3_) sandblasting after sintering. (e) Magnification 3000x, Al_2_O_3_ sandblasting after sintering; (f) magnification 3000x, CoJet (SiO_2_-coated Al_2_O_3_) sandblasting after sintering.

In order to compare the surface changes after mechanical pretreatment, zirconia before sintering, zirconia after sintering, the AL group and the Co group were all observed under a scanning electron microscope. Four zirconia blocks were coated with gold-palladium measuring 1 nm in thickness using Ion Sputter (JFC-1100E, JEOL, Japan). Images at 1000x and 3000x magnification were acquired using a scanning electron microscope (TM-1000, Hitachi, Japan) (Figure 5).

### 2.8. Statistical Analysis

Continuous variables are expressed as means and standard deviation, and categorical variables are presented as frequency and percentage. In order to compare between groups, we performed the one-way analysis of variance for shear bond strength and chi-square test (Fisher's exact test) for modified ARI results as appropriate. We analyzed multiple comparisons using Bonferroni correction and Tukey method, respectively. *p* value less than 0.05 was considered statistically significant and all statistical analyses were conducted using SPSS 21 version (IBM Co., Armonk, NY, USA).

## 3. Results

### 3.1. Shear Bond Strength

The results of shear bond testing are shown in Table 5.

Within the AL group, the SIL group showed the lowest bond strength (11.4 MPa), and the SBU group showed the highest (22.9 MPa) (*p* < 0.05). Within the SIL group, the AL group showed the lowest bond strength, with the CO group having significantly higher (*p* < 0.05) bonding strength. After thermocycling, within the AL group, the bond strength of the SIL group increased and that of the ZPP and SBU groups decreased. Within the CO group, the bond strength in all primer groups (SIL, ZPP, and SBU) increased after thermocycling. There was no statistically significant difference in bond strength after thermocycling between all groups (*p* > 0.05).

### 3.2. Fracture Pattern Analysis

The fracture pattern was examined under a stereomicroscope and was scored according to modified ARI (Table 4). The results are shown in Table 6. Representative images from the 12 groups are shown in Figure 3 (12x magnification).

All bond failures occurred between the zirconia and the resin adhesive. In the SIL groups, the CO group had significantly more resin remaining on the zirconia surface than did the AL group (*p* < 0.05). Conversely, in the ZPP and SBU groups, the AL group had more resin remaining on the zirconia surface than did the CO group. After thermocycling, the amount of resin remaining tended to decrease.

### 3.3. Changes after Surface Treatment on Zirconia

Under 1000x magnification, the zirconia surface after processing by the manufacturer but before sintering, there were marked lines on the surface. After sintering, the surface exhibited less marked lines and showed finer lines with homogenous appearance (Figures 5(a) and 5(b)). After sandblasting with Al_2_O_3_, scratches with sharp cracks were seen, and when sandblasted with CoJet, the changed surface coated with silica was observed (Figures 5(c)–5(f)). The changed surface and silica coating were even more evident in 3000x magnification (Figures 5(e) and 5(f)).

## 4. Discussion

**Figure 6:**
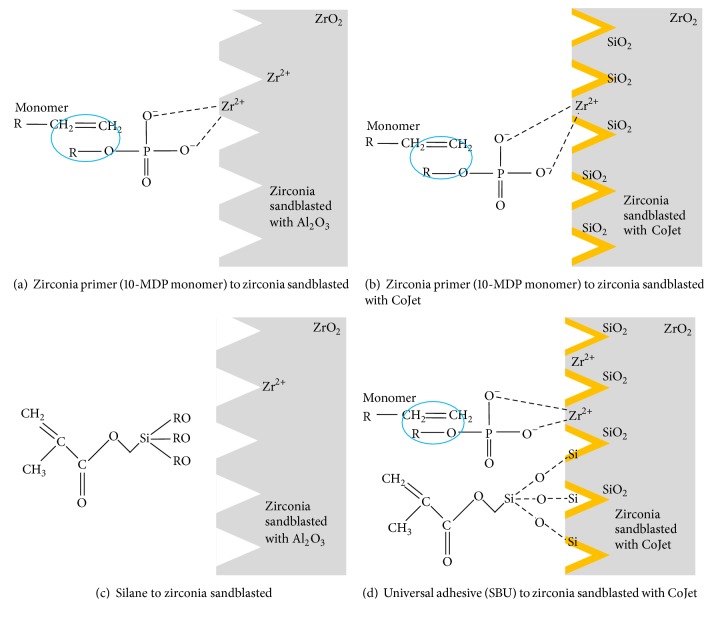
Mechanical and chemical interaction model about combined effect. (a) AL-ZPP: zirconia primer (10-MDP monomer) applied to zirconia with increased surface area by sandblasting with Al_2_O_3_; (b) CO-ZPP: zirconia primer applied to zirconia with less compatible surface after sandblasting by CoJet; (c) AL-SIL: silane primer applied to zirconia sandblasted with Al_2_O_3_; and (d) CO-SBU: Single Bond Universal applied to zirconia sandblasted with CoJet.

Zirconia as a restorative material has superior esthetics and durability [14], but in regard to its bond strength with orthodontic brackets, it is inferior to the bond between metal brackets and tooth enamel. Previous studies evaluated the effect of sandblasting and primers on shear bond strength [24–26]. This study investigated the combined effects of mechanical and chemical pretreatment on zirconia surface before bonding. Two sandblasting materials (CoJet and aluminum oxide) were used to increase the surface area for microretentive bonding, and three primers (silane as a porcelain primer, Zirconia Prime Plus as a zirconia primer, and a universal bond containing MDP and silane) were additionally used to induce chemical bonding. The expected outcome of these pretreatments are the following: (i) the increase in surface area after mechanical sandblasting (Figure 6), (ii) the chemical bonding between zirconia and 10-MDP containing primer (Figures 6(a), 6(b), and 6(d)), and (iii) siloxane bond between CoJet treated zirconia surface and silane-containing primer (Figures 6(c) and 6(d)). However, there has not been any study that investigated the effect of mechanical surface treatment and chemical primers at the same time. Therefore, in order to find the best combination of mechanical and chemical pretreatment under conditions best simulating the oral environment, different factors, sandblasting (AL/CO), primer (SIL/ZPP/SBU), and thermocycling (T/N), were studied (Figure 6).

The CO-SBU combination which combines all of the three effects showed the highest bond strength after thermocycling (Table 5, Figure 6(d)). CO-SIL, which has the combined effect of (i) and (iii), showed higher SBS than AL-SIL, which has the effect of only (i) (Table 5, Figure 6(c)). In addition, when comparing AL-ZPP (Figure 6(a)) and CO-ZPP (Figure 6(b)), which shows the combined effect of (i) and (ii), CO-ZPP resulted in lower bond strength than AL-ZPP before thermocycling as the zirconia surface that binds with ZPP was partially covered by silica coating after CoJet treatment, and ZPP alone does not contain silane that is able to bond with SiO_2_. In contrast, the CO group had higher bond strength than the AL group when either silane-containing primer (SIL or SBU) was applied [27]. The shear bond strength result range was comparable to those of previous studies [21, 22].

ZPP and SBU are primers that contain MDP, which is known to chemically bond with zirconia. They show high bond strength after surface roughening through sandblasting. Our results show a high shear bond strength even in CoJet treated, silica-coated zirconia. This probably means that the silica coating does not cover enough of the zirconia surface to inhibit bonding with MDP. Rather CoJet increases the surface area and partially covers the zirconia surface, promoting bonding with MDP. This is consistent with previous studies where silica-coated zirconia showed high bond strength after treatment with a primer containing MDP [24–26].

The shear bond strength after sandblasting and primer treatment did not decrease after thermocycling. Previous studies showing similar or increased bond strength after thermocycling [24–26, 28–30] demonstrated that when MDP primer was applied after silica coating, the shear bond strength increased with thermocycling, possibly due to residual polymerization of the adhesive [26]. Another study also showed that after tribochemical silica coating was applied to a ceramic surface, the shear bond strength was higher after thermocycling [25]. The stress of sandblasting on zirconia may cause phase transition and consequent volume expansion and internal compressive stress formation. This may result in strengthening of the zirconia through inhibition of microcracks. Sandblasting may have caused internal stress, and repeated expansion and shrinkage through thermocycling may have strengthened the zirconia and resulted in increased shear bond strength after thermocycling [5, 9]. This needs to be clarified through further studies.

The modified ARI results support the shear bond strength values we obtained (Table 6, Figure 4). The direction and magnitude of forces applied to orthodontic metal brackets after bonding to zirconia are different from the forces applied to cemented zirconia restorations. In addition, after the completion of orthodontic treatment, the brackets need to be removed without causing damage to the restoration surface. Therefore, the failure pattern has to be in the adhesive used. If the bond interface is weak, failure occurs at the interface, but if the bond is strong, similar in strength to resin, then the failure pattern is cohesive failure within the resin itself [31]. Therefore, it is clinically important to study the facture pattern and shear bond strength between resin cement and zirconia in metal bracket bonding. In this experiment, all bond failures occurred within the resin between the zirconia and the metal brackets (Figure 4). Both mechanical and chemical pretreatment increased the bond strength between zirconia and the resin adhesive. The CoJet and silane treatment group showed high bond strength and there was a large of amount of resin remaining on the zirconia surface (*p* < 0.05, Figures 4(a)–4(d)). In the AL-ZPP group, thermocycling decreased the shear bond strength. This is consistent with the M-ARI results as the amount of resin remaining on the zirconia surface decreased after thermocycling (Figures 4(e)–4(h)). In the SBU groups, much of the resin remained on the zirconia surface after thermocycling, but as with the shear bond strength results, there was no statistically significant difference (*p* > 0.05).

The SEM images were taken to examine the change in zirconia surface before sintering, after sintering, and after sandblasting by two different materials, Al_2_O_3_ and CoJet. Unlike other dental materials, zirconia has a crystalline structure change from stable monoclinic crystalline structure to tetragonal phase through phase transition when sintered at 1500°C [8]. Therefore, in order to examine the zirconia surface changes after such phase transition by sintering and stress formation through sandblasting, SEM images were obtained as in previous studies (Figure 5) [21, 22, 32]. When zirconia is placed under stress such as sandblasting or thermocycling, phase transformation from tetragonal phase to monoclinic phase occurs. This causes volume expansion, which stops the propagation of cracks, and results in higher flexural and fracture strength through transformation toughening [8]. Therefore, after phase transition through sintering, the zirconia surface showed a more homogeneous surface (Figures 5(a) and 5(b)), and after sandblasting the surface showed scratches and pits (Figures 5(c) and 5(e)). When the zirconia surface was sandblasted with CoJet, which forms a silica coating, the surface was markedly different from the surface sandblasted with Al_2_O_3_ (Figures 5(d) and 5(f)). These SEM results are consistent with previous studies showing SEM images after sandblasting [22]. Such increased surface area increases the SBS through micromechanical bond formation, as well as chemical bond formation with the chemical primer (Figure 6). Moreover, the SBS of this micromechanical bond may be increased through transformation toughening when additional stress is induced by thermocycling.

A limitation of the study is the lack of a negative control group, but the study was designed based on previous zirconia in vitro studies in the literature [5, 21, 22] and on our own pilot studies. As expected, zirconia showed increased shear bond strength after mechanical and chemical bonding. Even the shear bond strength of the AL-SIL-N group (11.4 MPa), which measured the lowest, was clinically acceptable as it was greater than 10 MPa [6, 7, 21]. Therefore, further studies evaluating clinically acceptable shear bond strength values are necessary so that specific recommendations regarding zirconia surface treatment methods may be provided. The number of cycles to be performed during thermocycling is controversial, but some claim that the exact number of cycles is not important as the effects are already prominent during the initial phases [33]. However, some studies claim the effects of thermocycling increase as the number of cycles increases [10, 34]. In this study, considering the phase transition, which occurs after thermocycling, 2000 cycles were selected. While the SEM images showed the changes in zirconia surface after mechanical treatment, the roughness was not quantified. In future studies, the measurement of roughness would help evaluate the surface change after mechanical treatment.

## 5. Conclusion

CO-SBU, which combines the effect of increased surface area by mechanical treatment and chemical bonding with both 10-MDP and silane, showed the highest shear bond strength. Sandblasting with either AL or CO improved the mechanical bonding by increasing the surface area, and all primer groups showed clinically acceptable increase of shear bond strength for orthodontic treatment.

## Figures and Tables

**Table 1:** Classification of experimental groups.

Groups	No thermocycling		Thermocycling	
	Al_2_O_3_	CoJet	Al_2_O_3_	CoJet
Silane	AL-SIL-N	CO-SIL-N	AL-SIL-T	CO-SIL-T
Zirconia Prime Plus	AL-ZPP-N	CO-ZPP-N	AL-ZPP-T	CO-ZPP-T
Single Bond Universal	AL-SBU-N	CO-SBU-N	AL-SBU-T	CO-SBU-T

**Table 2:** Surface treatment product details of zirconia specimens.

	Materials	Products	Manufacturer	
Mechanical method	Al_2_O_3_	Blasting Medium for crowns and bridges (LOT#444262)	Dentaurum	Ispringen, Germany
	Silica-coated Al_2_O_3_	CoJet (LOT#574170)	3M ESPE	St. Paul, MN, USA

Chemical method	Silane coupling agent	Reliance Porcelain Conditioner (LOT#148214)	Reliance Orthodontic Products, Inc.	Itasca, IL, USA
	Zirconia primer	Zirconia Prime Plus (LOT#1600000977)	Bisco, Inc.	Schaumburg, IL, USA
	Universal bond	Single Bond Universal (LOT#615486)	3M ESPE	St. Paul, MN, USA

**Table 3:** Composition of primers applied to zirconia specimens.

Trade name	Functional monomer	Manufacturer	
Porcelain Conditioner	Silane	Reliance	Itasca, IL, USA
		Orthodontic	
		Products,	
		Inc.	

*Z*-Prime Plus	Organophosphate and carboxylic acid, biphenyl dimethacrylate and hydroxyethyl methacrylate, MDP	Bisco, Inc.	Schaumburg, IL, USA

Single Bond Universal	MDP, Bis-GMA, HEMA, ethanol, water, silane treated silica, 2-propenoic acid, 2-methyl-, reaction products with 1,10-decanediol and phosphorous oxide, copolymer of acrylic and itaconic acid, dimethylaminobenzoate(-4), camphorquinone, 2-(dimethylamino)ethyl methacrylate, methyl ethyl ketone	3M ESPE	St. Paul, MN, USA

MDP: methacryloyloxydecyl dihydrogen phosphate; Bis-GMA: bisphenol A-glycidyl methacrylate; HEMA: hydroxyethylmethacrylate.

**Table 4:** Modified Adhesive Remnant Index (ARI) scoring method.

ARI Score	Criteria
1	All of the composite remained on zirconia
2	More than 90% of the composite remained on zirconia
3	More than 10% but less than 90% of the composite remained on zirconia
4	Less than 10% of the composite remained on zirconia
5	No composite remained on zirconia

**Table 5:** Comparison of shear bond strengths (mean ± standard deviation) by sandblasting and primer, thermocycling groups (MPa).

	Primers	No thermocycling	Thermocycling
Al_2_O_3_	Silane	11.4 ± 5.8^a,b^	13.7 ± 5.0^a,b,c^
	ZPP	21.6 ± 3.3^d,e^	20.0 ± 4.9^c,d,e^
	SBU	22.9 ± 6.5^d,e^	22.5 ± 6.9^d,e^

CoJet	Silane	19.7 ± 4.1^c,d,e^	25.0 ± 5.0^e^
	ZPP	20.5 ± 5.4^c,d,e^	24.1 ± 3.5^d,e^
	SBU	24.2 ± 2.8^d,e^	26.2 ± 3.1^e^

Different superscripts denote significant differences between groups not sharing the same superscript (*p* < 0.05).

**Table 6:** Modified Adhesive Remnant Index results.

Groups (*n* = 10)	Modified Adhesive Remnant Index					*p* value
	1	2	3	4	5	
AL-SIL-N			1 (10%)	6 (60%)	3 (30%)	<0.0001
AL-SIL-T				7 (70%)	3 (30%)	
CO-SIL-N^*∗*†^	8 (80%)	1 (10%)	1 (10%)			
CO-SIL-T^*∗*†^	7 (70%)	2 (20%)	1 (10%)			

AL-ZPP-N	6 (60%)		2 (20%)	1 (10%)	1 (10%)	0.037
AL-ZPP-T		1 (10%)	5 (50%)	3 (30%)	1 (10%)	
CO-ZPP-N	1 (10%)		3 (30%)	2 (20%)	4 (40%)	
CO-ZPP-T		1 (10%)	1 (10%)	6 (60%)	2 (20%)	

AL-SBU-N	3 (30%)	4 (40%)	3 (30%)			0.100
AL-SBU-T	6 (60%)	1 (10%)	2 (20%)	1 (10%)		
CO-SBU-N		2 (20%)	4 (40%)	4 (40%)		
CO-SBU-T	4 (40%)		2 (20%)	2 (20%)	2 (20%)	

^*∗*^ *p* < 0.05 versus AL-SIL-N; ^†^*p* < 0.05 versus AL-SIL-T.
